# Dietary guanidinoacetic acid supplementation improves rumen metabolism, duodenal nutrient flux, and growth performance in lambs

**DOI:** 10.3389/fvets.2025.1528861

**Published:** 2025-02-06

**Authors:** Shiqi Zhang, Mireguli Yimamu, Chen Ma, Jun Pan, Caidie Wang, Wenjie Cai, Kailun Yang

**Affiliations:** College of Animal Science, Xinjiang Agricultural University, Urumqi, China

**Keywords:** lambs, guanidinoacetic acid, creatine, rumen metabolism, ruminal escape rate

## Abstract

Guanidinoacetic acid (GAA) is the only precursor of creatine, which is an important energy source for growth and metabolism. The degradation of guanidinoacetic acid in rumen plays a decisive role in its application in ruminant diet. Two experiments were conducted to investigate the rumen GAA escape rate and its effects on metabolism, blood metabolites and growth performance of Kazakh male lambs. In the first part of the experiment, 24 Kazakh male lambs equipped with rumen and duodenal fistulas were selected to determine the rumen escape rate of guanidylacetic acid. In the second part, 24 healthy Kazakh male lambs were selected to determine the growth performance. They were divided into 4 groups and fed a basal diet supplemented with 0, 500, 1,000, 1,500 mg/kg GAA, respectively. The results show that ruminal escape rates of 36–56% were achieved in lambs with dietary GAA supplementation at 500–1,500 mg/kg DM. Dietary 1,500 mg/kg DM GAA increased levels of creatine, IGF-I and insulin, and promoted lamb growth.

## 1 Introduction

Guanidinoacetic acid (GAA) is the only precursor of creatine, which is an important energy source for growth and metabolism in vertebrates. The addition of exogenous GAA stimulates creatine biosynthesis (1). Although creatine levels can be increased by direct dietary supplementation, GAA is a more stable and potent inducer of creatine synthesis (2). The addition of GAA to animal diets has been shown to promote muscle energy metabolism and growth performance (3–5). For example, dietary GAA supplementation can improve carcass quality, energy metabolism and meat quality in fattening pigs (6) and promote growth performance in broilers (7–9). Furthermore, the metabolites produced (creatine, creatine phosphate, creatinine) as well as unmetabolized GAA are excreted in the urine, with previous studies confirming that no harmful residues are detected in livestock products (10).

Several studies on GAA supplementation in cattle via abomasum continuous infusion (11) showed increased plasma and urinary creatine concentrations and enhanced creatine synthesis (12). These findings suggest that GAA can act as a precursor and stimulate creatine synthesis in cattle. A previous study showed that the bioavailability of exogenous GAA after infusion into the rumen and abomasum of cattle was approximately 50%, indicating that GAA is degraded in the rumen (13); however, specific degradation rates and changes in nutrient flow were not reported in detail. GAA degraded in the rumen may be used by microorganisms to support their growth. Furthermore, Li et al. (14) reported that the addition of 0.6 or 0.9 g/kg DM GAA improved growth performance, nutrient digestion and rumen fermentation in Angus beef cattle, although the escape rate of dietary GAA in the rumen and the effect on rumen fermentation remain to be clarified.

As an animal-derived amino acid derivative, GAA is largely absent from whole plant protein-based lamb rations. The effects of GAA on growth performance and rumen metabolism in sheep have not been reported. To bridge a current knowledge gap, we investigated the extent to which GAA added exogenously in the diet is degraded in the rumen and its effects on rumen metabolism and growth performance in lambs.

## 2 Materials and methods

All experimental procedures involving animals were approved (animal protocol number: 2020024) by the Animal Welfare and Ethics Committee of Xinjiang Agricultural University, Urumqi, Xinjiang, China.

### 2.1 Experimental materials

GAA (non-rumen protected; purity >98%) was purchased from Genetech Biotechnology Co., Ltd. (Beijing, China).

All lambs used in this study were commercial livestock purchased from a local market and owned by this research team. Before study, the purchased lambs were quarantined and cared by Huikang Animal Husbandry Co., Ltd. (Changji, Xinjiang). After the study, all the lambs were euthanized by carotid bleeding under anesthesia (i.v. Lumianning, main component xylazine hydrochloride, 20 mg/kg BW [injected]) and then treated following harmless disposal procedures.

### 2.2 Experimental design

Experimental design 1: To investigate the effect of supplemental GAA on ruminal GAA flow rate and rumen metabolism in lambs, 24 Kazakh rams [aged 5 months, weight (34.29 ± 1.95) kg] with ruminal and proximal duodenal fistulas were selected. The test lambs (*n* = 6 per group) were fed a basal diet (without creatine and GAA) supplemented with GAA at 0 mg (0 mg/kg group), 500 mg (500 mg/kg group), 1,000 mg (1,000 mg/kg group) and 1,500 mg (1,500 mg/kg group) per kg of dry matter (DM) basal diet. A non-isotopic marker (Li, Cr-EDTA) was used to determine the chyme flow rate. The experiment lasted for 23 days, 15 days of adaptation and 8 days of sampling.

Experimental design 2: We then investigated the effect of dietary GAA supplementation on blood metabolites and growth performance in 24 randomly selected healthy Kazakh rams (aged 3 months, (27.35 ± 0.58) kg). The test lambs (*n* = 6 per group) were fed a basal diet (without creatine and GAA) supplemented with GAA at 0 mg (0 mg/kg group), 500 mg (500 mg/kg group), 1,000 mg (1,000 mg/kg group) and 1,500 mg (1,500 mg/kg group) per kg of DM basal diet. The feeding trial was conducted for 55 d consisting of a 10-day pre-feeding period and a 45-day trial period.

The basal diet was formulated according to the NRC (2007) nutritional requirements. The diet formulations and nutrient levels are shown in Table 1.

**Table 1:** Feed ingredients and nutrient levels of experimental diets (%).

Ingredient	Ratio
Maize	30.00
Wheat bran	7.20
Soybean meal	12.00
Cottonseed meal	7.80
Alfalfa hay	20.00
Wheat straw	20.00
Premix^A^	3.00
Total	100

Items	Nutrient levels (DM basis)
Organic matter (OM)	89.87
Crude protein (CP)	16.58
Ether extract (EE)	1.53
Neutral detergent fiber (NDF)	46.16
Acid detergent fiber (ADF)	32.62
Calcium (Ca)	0.58
Phosphorus (P)	0.33
Lysine	0.81
Methionine + cysteine	0.58

^A^The premix provided the following per kg of the concentrate supplement: Vitamin A 10,000.00 IU, Vitamin D3 2,550.00 IU, Vitamin E 20.00 IU, niacin 20.00 mg, biotin 0.06 mg, Cu (as copper sulfate) 22.00 mg, Fe (as ferrous sulfate) 94 mg, Mn (as manganese sulfate) 80 mg, Zn (as zinc sulfate) 88 mg, I (as potassium iodide) 0.75 mg, Se (as sodium selenite) 0.5 mg, Co (as cobalt chloride) 0.33 mg, Ca (as calcium carbonate and calcium hydrogen phosphate) 0.35%, P (as calcium hydrogen phosphate) 0.125%, NaCl 0.8%.

### 2.3 Feeding management

The test lambs were numbered and housed in semi-open sheds (1.2 m × 1.5 m) with good ventilation. The lambs were fed daily in two separate feedings at 08:00 and 20:00. GAA was mixed into the feed concentrate. Lambs were fed and watered ad libitum. Feed intake was recorded daily and each lamb was weighed every 15 days. Dry matter intake (DMI), average daily gain (ADG) and feed weight ratio (F:G) were calculated for each lamb. Disinfection and sterilization of the facility and immunization of the animals were conducted according to routine farm procedures.

### 2.4 Sample collection and processing

In experiment 1, ruminal fluid was collected on day 16 of the trial period at 0 h (before feeding) and 1, 2, 4, 6 and 8 h after the morning feed, filtered through nylon cloth and then frozen. Duodenal chyme (chyme is a mixture and includes both solids and liquids) were collected in three batches (8 lambs per time-point, 2 lambs per treatment group) on days 17–23 of the trial period according to the disposable perfusion method (15). In brief, samples of duodenal chyme were collected from the test lambs prior to perfusion to determine background values. Each lamb received a perfusion of Chromium-EDTA(Cr-EDTA, 12 g/lamb) delivered using a 100-mL syringe fitted with a fine tube (approx. 20 cm) to facilitate even dispersion to different parts of the rumen. After the perfusion, duodenal chyme samples (20 mL) were collected at 0 h (before the morning feed), and 1, 2, 3, 4, 6, 8, 12, 16, 24, 32, 40 and 48 h, after feeding. At each time-point, 5 g of ruminal or duodenal chyme samples were mixed to produce a mixed sample for the determination of DM, GAA, total nitrogen and total reducing sugar content.

In experiment 2, blood was collected from the jugular vein of lambs at 0 h before the morning feed on day 45 of the test period. The blood was centrifuged at 3,500 × *g* for 15 min to isolate the plasma, which was stored at −20°C.

### 2.5 Sample analysis and calculations

The diet samples were finely ground, passed through a 1-mm mesh (Thomas-Wiley Laboratory Mill Model 4, Thomas Scientific, Swedesboro, NJ, USA), and analyzed for the contents of dry matter (DM), organic matter (OM), crude protein (CP), fat (EE), acid detergent fibers (ADF), and neutral detergent fibers (NDF). The samples (5.0 g) were dried at 105°C overnight according to the AOAC method (16). The nitrogen content was determined using a nitrogen analyzer combustion method (990.03; AOAC) (Model CNS-2000; LECO Company, St.1990), and CP was calculated as N× 6.25. Ether-like hexane extracts were obtained from the samples using the Ankom Extraction System (Macedonia, New York). The NDF and ADF contents were determined as described by Van Soest et al. (17) and Goering et al. (18). After anerobic hydrolysis, the amino acid content of the diet samples was analyzed using a Sykam S433D Amino Acid Analyzer (Sykam, Germany).

The rumen and duodenal chyme collected were air-dried in an oven at 60°C and then subjected to DM determination at 105°C. The chromium concentration was determined by inductively coupled plasma mass spectrometry (THERMO, USA, model iCAPQ) using the DM of the rumen and duodenal chyme as described above. Method: The sample was weighed into a PTFE digestion tank and 5 mL of nitric acid was added. The sample was left to stand, sealed and placed in a microwave digestion apparatus. After the temperature had cooled to <50°C, the digestion tank was removed and placed in a fume hood; blanks were treated in the same way. Total nitrogen in the contents of the rumen and duodenum was determined using the Kjeldahl method (16). Total reducing sugars were determined by the tetrazolium blue chloride method (19).

The DM of duodenal chyme was weighed and 500 mg was dissolved in 1 mL of distilled water, extracted by ultrasonication for 10 min and centrifuged at 12,000 × *g* for 15 min. The supernatant was then removed for determination of GAA. Ruminal fluid samples were filtered through nylon mesh and centrifuged at 12,000 × *g* for 15 min. The supernatant was then removed for determination of creatine and GAA. The concentrations of GAA and Cr in the supernatants were determined according to methods described by Wada et al. (20). Briefly, to precipitate the protein, 200 μL plasma or supernatant was mixed with 400 μL acetonitrile and incubated for 10 min before centrifugation at 12,000 × *g* for 10 min. Subsequently, 100 μL clear supernatant was mixed with 200 μL phosphoric acid (2 mM). A sample of the mixture (20 μL) was then analyzed using an IC YS-50 weak acid cation exchange column (4.6 mm × 125 mm) under the following conditions: flow rate, 1.0 mL/min; column temperature, 30°C; detection wavelength, 210 nm; elution mode, one-time linear elution.

Ruminal fluid pH was determined using a PE20K Mettler Toledo (METTLER TOLEDO, Shanghai, China) acidity meter.

Volatile fatty acids (VFA) were determined by gas chromatography using 4-methyl-N-valeric acid as an internal standard. Ammoniacal nitrogen (NH_3_-N) was determined using an alkaline sodium hypochlorite-phenol spectrophotometric method (21). Protozoa counts were determined using a hemocytometer after adding formaldehyde solution (37% formaldehyde (v/v): 0.9% (w/v) NaCl, 1:9) (22).

Plasma levels of insulin (INS), growth hormone (GH) and insulin growth factor-I (IGF-I) were determined in lambs 0 h before the morning feed on day 45 of the trial by immunoradiometric assay using enzyme-linked immunosorbent assay (ELISA) kits (Nanjing Jiancheng Bioengineering Institute, Nanjing, China).

### 2.6 Duodenal inflow calculations

The duodenal inflow was calculated after first matching the decreasing curve of chromium concentration in DM per unit of rumen chyme with sampling time according to Equation 1:


(1) $$C=C_{0}\times e^{-kt}$$

where *C* = the concentration of Cr in the duodenal chyme sample, *C_0_* = the concentration of Cr in the duodenal chyme sample at *t* = 0, *k* & *t* = the dilution constants and the sampling time, respectively.

The fitted equations for duodenal inflow obtained from the determination of chyme chromium concentration in experiment 1 are shown in Table 2.

**Table 2:** Fitting equations for duodenal chyme chromium concentrations.

Group	C_0_	*k*	Exponential equation	*R*^2^
0 mg/kg	6.8495	0.0885	y = 6.8495e*^-0.0885x^*	0.9864
500 mg/kg	6.0481	0.0827	y = 6.0481e*^-0.0827x^*	0.9911
1,000 mg/kg	6.5505	0.0737	y = 6.5505e*^-0.0737x^*	0.9906
1,500 mg/kg	6.6911	0.0652	y = 6.6911e*^-0.0652x^*	0.9799

Then, the duodenal inflow volume of the rumen chyme was then calculated using Equation 2:


(2) $$Qr=\frac{Cr}{C_{0}}$$

Where Qr = duodenal inflow volume (kg/day), Cr = perfusion dose of chromium in the test lambs (mg), C_0_ = the concentration of chromium in the duodenal chyme sample at *t* = 0.

The GAA, total nitrogen and total reducing sugars of duodenal inflow are the multiplications of the GAA, total nitrogen and total reducing sugars concentration values of the mixed duodenal chyme samples and the duodenal inflow values.

### 2.7 Data analysis

Ruminal fluid pH, VFA, ammonia nitrogen and protozoa counts were calculated using a MIXED model with repeated observations in SAS (Version 9.2; SAS Inst. Inc., Cary, NC, USA). The fixed effects were the treatment (Trt), the sampling time-point (Date) and the interaction between the two (Trt × Date). The interaction between treatment and date was primarily analyzed in terms of its effects on test treatment. Other indicators analyzed using the GLM program of SAS (Version 9.2; SAS Inst. Inc., Cary, NC, USA). Orthogonal polynomial comparison coefficients were used to determine the linear and quadratic effects of the elevated dietary GAA levels on different parameters. The results were presented as least squares means and standard error of mean (SEM), with *p* < 0.05 set as the threshold for statistical significance.

## 3 Results

### 3.1 Changes in the level of GAA in the ruminal fluid of lambs

No GAA was detected in the ruminal fluid of lambs in the 0 mg/kg group. Complete ruminal evacuation of GAA was completed after 8 h of dietary GAA supplementation in the 500 mg/kg group, and within 12 h in the 1,000 mg/kg group. In the 1,500 mg/kg group, ruminal evacuation of GAA was incomplete after 12 h of dietary GAA supplementation (Table 3).

**Table 3:** Effect of dietary guanidinoacetic acid supplementation on the guanidinoacetic acid content of rumen fluid in lambs (μmol/L).

Item	Dietary GAA supplement levels, mg per kg DM diet				SEM^A^
	0	500	1,000	1,500	
0 h	–^B^	–	–	118.24	6.68
1 h	–	406.11	827.67	1,315.81	23.43
2 h	–	296.39	612.38	1,076.44	28.91
4 h	–	133.62	328.34	705.79	18.58
6 h	–	72.26	210.10	495.01	20.26
8 h	–	–	136.52	240.69	17.39

^ASEM = standard error of the least squares means; n = 6 lambs/group.^

B“–” means that the concentration was not detected under this test condition.

### 3.2 Ruminal fermentation parameters

Compared to the 0 mg/kg group, the pH of the rumen fluid was significantly higher in the 1,500 mg/kg group (Treatment, *p* < 0.05) (Table 4). Dietary GAA supplementation at 500–1,500 mg/kg increased the ruminal ammonia content in lambs (Treatment, *p* < 0.05), and there was a significant interaction between date and treatment (Interaction, *p* < 0.05). Propionic acid, butyric acid and isovaleric acid levels were significantly lower in the 500 mg/kg group compared to those in the 0 mg/kg group (Treatment, *p* < 0.05). Propionic acid levels were significantly lower in the 1,500 mg/kg group and butyric acid levels were significantly higher than those in the 0 mg/kg group (Treatment, *p* < 0.05). The Acetic acid: propionic acid ratio was significantly higher in the 500 mg/kg and 1,500 mg/kg groups than that in the 0 mg/kg group (Treatment, *p* < 0.05). The protozoa counts were significantly higher in the sheep rumen fluid of the 1,500 mg/kg group compared to that in the 0 mg/kg group (Treatment, *p* < 0.05), and there was a significant interaction between date and treatment (Interaction, *p* < 0.05).

**Table 4:** Effects of dietary guanidinoacetic acid supplementation on ruminal fermentation parameters in lambs.

Item	Dietary GAA supplement levels, mg·kg^−1^				SEM^A^	*p*-value		
	0	500	1,000	1,500		Treatment	Date	Interaction
pH	6.32^b^	6.31^b^	6.44^ab^	6.52^a^	0.03	<0.01	<0.01	0.95
NH_3_-N, mg·100 mL^−1^	21.33^b^	25.84^a^	24.96^a^	24.53^a^	0.47	<0.01	<0.01	<0.01
Total olatile fatty acids, mmol·L^−1^	81.51	74.18	77.56	79.34	1.99	0.72	<0.01	0.28
Acetate, %	62.35^a^	65.87^b^	62.79^a^	63.60^b^	1.23	0.01	<0.01	0.16
Propionate, %	20.62^b^	17.93^c^	20.98^bc^	16.49^a^	0.23	<0.01	<0.01	0.84
Butyrate, %	14.02	13.14	13.05	16.54	0.32	0.21	<0.01	0.49
Isobutyrate, %	0.98	1.09	1.12	1.21	0.05	0.93	<0.01	0.25
Valerate, %	0.87	1.00	1.01	0.89	0.07	0.03	0.06	0.76
Isovalerate, %	1.15^a^	0.98^b^	1.10^ab^	1.27^a^	0.06	0.35	<0.01	0.30
A:P^B^	3.31^b^	3.88^a^	3.20^b^	4.20^a^	0.15	<0.01	<0.01	0.06
Protozoa, ×10^5^/mL	2.76^b^	2.88^ab^	2.92^ab^	3.01^a^	0.92	0.11	<0.01	<0.01

^A^SEM = standard error of the least squares means (*n* = 6 lambs/group).

^BA:P = acetate: propionate.^

^a–c^Least squares mean values within a row with different superscripts differed (*p* < 0.05).

### 3.3 Nutrition duodenal inflow

There were no significant differences in the DMI and duodenal inflow (DM) between the groups (Linear & Quadratic, *p* > 0.05, Table 5). No GAA was detected in the duodenal chyme of lambs in the 0 mg/kg group. The GAA flux in the duodenal chyme of lambs in the 500 mg/kg, 1,000 mg/kg and 1,500 mg/kg groups reached 393.78 mg/d, 451.55 mg/d and 655.37 mg/d, respectively, and the ruminal escape rates reached 65.63, 37.63 and 36.41%, respectively. As dietary GAA levels increased, duodenal reducing sugar influx increased linearly. The amount of total reducing sugars entering the duodenum of lambs was significantly higher in the 1,000 mg/kg and 1,500 mg/kg groups compared to that in the 0 mg/kg group (Linear, *p* < 0.05). There were no significant differences in the total nitrogen entering the duodenum between the groups (Linear & Quadratic, *p* > 0.05).

**Table 5:** Effects of dietary guanidinoacetic acid supplementation on the nutrition duodenal inflow in lambs.

Item	GAA supplement levels, mg·kg^−1^ DM diet				SEM ^A^	*p*-value	
	0	500	1,000	1,500		Linear	Quadratic
DMI, g/day	1,096.24	1,081.64	1,096.31	1,087.96	11.36	0.84	0.79
Duodenal inflow (DM), g/day	341.67	361.98	352.44	354.25	19.63	0.75	0.64
GAA ruminal escape, mg/day	–^B^	393.78	451.55	655.37	28.75		
GAA percentage ruminal escape, %	–	65.63	37.63	36.41	3.37		
Total reducing sugar flow flux, mg/day	405.99	520.83	568.25	590.48	41.64	< 0.01	0.28
Total nitrogen flow flux, g/day	16.20	16.51	15.58	16.37	0.95	0.92	0.81

^ASEM = standard error of the least squares means; n = 6 lambs/group.^

^B“–” means that the concentration was not detected under this test condition.^

### 3.4 Jugular venous plasma parameters

With increasing levels of dietary GAA supplementation, jugular venous plasma GAA, creatine and IGF-I levels increased linearly (Linear, *p* < 0.05, Table 6) while glucose concentrations decreased linearly (Linear, *p* < 0.05).

**Table 6:** Effects of dietary guanidinoacetic acid supplementation on jugular vein plasma parameters in lambs.

Item^A^	GAA supplement levels, mg·kg^−1^ DM diet				SEM^B^	*p*-value	
	0	500	1,000	1,500		Linear	Quadratic
GAA, μmol/L	162.96	166.63	180.79	203.75	5.86	<0.01	0.24
Creatine, μmol/L	106.76	113.62	117.48	123.32	2.51	<0.01	0.90
Glucose, mmol/L	5.51	5.28	5.16	5.04	0.12	0.01	0.67
GH, ng/mL	1.88	2.05	1.92	1.69	0.11	0.18	0.09
IGF-I, ng/mL	99.90	114.08	100.14	135.35	7.69	0.02	0.19
INS, mIU/L	20.39	20.81	19.19	24.38	1.11	0.05	0.05

^AGH = growth hormone; IGF-I = insulin-like growth factor-1; INS = insulin.^

^BSEM = standard error of the least squares means; n = 6 lambs/group.^

### 3.5 Growth performance

The average day gain of lambs showed a linear increase with dietary GAA supplementation (Linear, *p* < 0.05, Table 7).

**Table 7:** Effects of supplementation with GAA on growth performance in lambs.

Item^A^	GAA supplement levels, mg·kg^−1^ DM diet				SEM^B^	*p*-value	
	0	500	1,000	1,500		Linear	Quadratic
Initial BW, kg	27.83	27.45	27.55	27.38	0.31	0.42	0.75
Day 45 BW, kg	33.95	33.92	34.09	34.72	0.42	0.15	0.52
DMI, g (DM)	901.42	897.45	899.57	913.78	7.14	0.23	0.22
AG, kg	6.12	6.47	6.54	7.34	0.38	0.04	0.57
ADG, g/d	135.93	143.78	145.33	163.04	8.48	0.04	0.57
F:G	6.90	6.37	6.26	5.64	0.44	0.06	0.92

^ABW = body weight; DMI = dry matter intake; AG = average gain; ADG = average day gain; F:G = feed to gain ratio.^

^BSEM = standard error of the least squares means; n = 6 lambs/group.^

The least squares means for BW, DMI, ADG, F:G in the table are the results of an analysis of covariance with initial weight as the covariate.

## 4 Discussion

In recent years, the potential benefits of dietary GAA supplementation in ruminants have become a focus of research. In this study, we investigated the ruminal escape of GAA in lambs and its impact on their growth performance. We showed that dietary GAA supplementation was associated with a ruminal escape rate of GAA ranging from 36 to 65%, and the average daily gain of lambs increased linearly with the supplementation of GAA in the diet.

The ruminal GAA escape rate ranged from 36 to 65%, indicating partial degradation of GAA in the rumen, which is consistent with the study reported by Speer et al. (13) showing that GAA bioavailability was approximately 50% after infusion into the rumen and wrinkled stomach of cattle. The inclusion of GAA in the diet facilitated its degradation in the rumen, causing an elevation in ammoniacal nitrogen content and an increase in the pH of the rumen fluid. This effect may be correlated with alterations in the protozoa population, particularly in groups receiving 1,000 mg/kg and 1,500 mg/kg of GAA, where their numbers increased. Notably, protozoa consume bacteria for nourishment (23, 24). The heightened protozoa population subsequently increased their consumption of bacteria and starch granules, resulting in a decline in bacterial count and reduced carbohydrate availability. This, in turn, impeded the degradation and utilization of both carbohydrates and proteins (25), further contributing to the increase in pH. Additionally, the expanded protozoa population ingested significant quantities of microbial proteins, thereby enhancing nitrogen excretion via the release of ammonia (26).

The increase in protozoa numbers in the rumen may be due to the use of GAA as a nitrogen source to provide energy and promote division. The nitrogen resources in the protozoa are derived partly from bacterial proteins obtained by engulfing bacteria, and partly from protein nitrogen supplied in the diet (27). Proteolytic enzymes are found in ruminal protozoa and are highly active, engulfing insoluble protein particles, free amino acids and bacteria to synthesize protozoal proteins (28, 29).

In this study, we did not observe a significant change in the flow of total duodenal nitrogen material. However, there was a linear increase in the flow of GAA into the small intestinal chyme, indicating an increase in the proportion of available nitrogen entering the small intestine; therefore, we hypothesized that dietary GAA improves nitrogen utilization in sheep. Adding 1,500 mg/kg GAA to lamb diets improved nitrogen retention in lambs and also demonstrated that dietary GAA can improve nitrogen utilization in sheep. In addition, the increase in the number of protozoa, which engulf starch granules and store them as branched chain starch that enters the small intestine with the chyme, increases the flow of reducing sugars from the small intestine chyme (27, 30). This phenomenon may also account for the increase in the flow of reducing sugars from the duodenum after dietary GAA supplementation and the improvement in the utilization of non-structural carbohydrates.

In this study, growth performance was improved lambs receiving 1,500 mg/kg MD GAA, with an increase in average daily weight gain and reduced average feed-to-weight ratio. Significantly elevated jugular vein plasma levels of creatine and GAA were observed with increasing dietary GAA supplementation, indicating incomplete degradation of GAA by ruminal microorganisms. Comparable findings were reported in cattle receiving rumen infusions of GAA (13). Speer et al. (13) stated that approximately 50% of GAA undergoes ruminal degradation, and rumen-infused GAA leads to increased plasma and urinary concentrations of Cr and enhanced Cr synthesis. Increasing creatine levels in animals can conserve the raw materials for protein synthesis and energy sources such as glycine and arginine, to improve energy metabolism, accelerate animal growth and increase feed utilization. Thus, the increase in creatine levels induced by the addition of GAA acid to the diet may be one of the mechanisms by which GAA improves growth performance.

Moreover, the addition of 1,500 mg/kg GAA to the diet increased plasma insulin levels and decreased plasma glucose levels. This may be explained by the presence of a positive charge on the GAA side-chain (31), which affects the depolarization of islet cell membranes, stimulating the secretion of protein kinase A and C-type insulin and improving the sensitivity of the mechanism that regulates insulin secretion (32). Previous studies have shown that exogenous GAA stimulates insulin secretion in rodents (33), and reduces plasma glucose levels (34). This, combined with the increased availability of reducing sugars in the duodenum, suggests that the addition of GAA to the diet increased the rate of glucose metabolism and facilitated glucose utilization, thus exerting a positive effect on the energy supply available to the organism for growth.

The addition of 1,500 mg/kg GAA to the diet increased plasma IGF-I levels, possibly by conserving arginine and stimulating IGF-I secretion. The addition of GAA to the diet of broiler chickens (8) also increased IGF-I secretion in the blood. IGF-I has been reported to improve amino acid utilization and increase net protein gain, thereby promoting body growth (35). Changes in IGF-I and insulin secretion may be one of the mechanisms by which dietary GAA supplementation improves the growth performance of lambs.

Looking ahead, the application of GAA in lamb production holds significant promise. The ability of GAA to enhance growth performance and nutrient utilization, as demonstrated in this study, suggests that it could become a valuable feed additive in the lamb industry. By improving average daily gain and feed efficiency, GAA has the potential to reduce the time required for lambs to reach market weight, thereby increasing the overall productivity and profitability of lamb farming operations. Furthermore, the positive effects of GAA on rumen fermentation and blood metabolites, such as increased microbial protein synthesis and elevated levels of IGF-1, indicate that it can support optimal health and development in lambs. This could lead to improved meat quality and yield, which are critical factors for consumer satisfaction and market competitiveness. Additionally, the synergistic potential of GAA with other feed additives, like betaine, offers an opportunity to further enhance its benefits. For instance, combining GAA with betaine has been shown to improve nutrient digestibility and energy-nitrogen metabolism in lambs, although the combined effect on growth performance may not be significantly greater than the individual effects of each additive. This highlights the importance of continued research to optimize the use of GAA in lamb diets and explore its interactions with other feed components. Moreover, long-term studies are needed to assess the sustained impact of GAA supplementation on physiological parameters and overall health of lambs over extended periods. By addressing these research gaps, the lamb industry can harness the full potential of GAA to drive advancements in production efficiency and animal welfare.

## 5 Conclusion

Ruminal GAA escape rates of 36–56% were achieved in lambs with dietary GAA supplementation at 500–1,500 mg/kg DM. Dietary supplementation of 1,500 mg/kg GAA increased the ruminal ammoniacal nitrogen concentration and the total reducing sugar flow into the small intestine, promoted the creatine level and glucose utilization in lambs, and increased daily weight gain in lambs.

## Data Availability

The original contributions presented in the study are included in the article/Supplementary material, further inquiries can be directed to the corresponding author.

## Supplementary material

SUPPLEMENTARY TABLE 1 Feed ingredients and nutrient levels of experimental diets (%).

SUPPLEMENTARY TABLE 2 Fitting equations for duodenal chyme chromium concentrations.

SUPPLEMENTARY TABLE 3 Effect of dietary guanidinoacetic acid supplementation on the guanidinoacetic acid content of rumen fluid in lambs.

SUPPLEMENTARY TABLE 4 Effects of dietary guanidinoacetic acid supplementation on ruminal fermentation parameters in lambs.

SUPPLEMENTARY TABLE 5 Effects of dietary guanidinoacetic acid supplementation on the nutrition duodenal inflow in lambs.

SUPPLEMENTARY TABLE 6 Effects of dietary guanidinoacetic acid supplementation on jugular vein plasma parameters in lambs.

SUPPLEMENTARY TABLE 7 Effects of supplementation with GAA on growth performance in lambs
